# An exploratory comparison of the short-term effects of two different static cold storage techniques of the graft in kidney transplantation from living donors

**DOI:** 10.1016/j.clinsp.2026.101061

**Published:** 2026-07-28

**Authors:** Murat Ferhat Ferhatoğlu, Taner Kivilcim, Sibel Y. Ferhatoglu, Alp Gurkan

**Affiliations:** a Department of Surgery, Medical School of Halic University, Istanbul, Turkey; b Department of Surgery, Medical School of Istanbul Okan University, Istanbul, Turkey; c Department of Anesthesiology and Reanimation at Dr. Siyami Ersek Thoracic and Cardiovascular Surgery Training and Research Hospital, Health Sciences University, Istanbul, Turkey

**Keywords:** Kidney, Transplantation, Static storage, Graft function, Ice bag, Raytech sponge

## Abstract

**Objective:**

Maintaining graft viability between extraction and implantation is critical for optimal graft function and short-term survival. Newer methods like normo/hypothermic machine perfusion have shown success in deceased donor transplants. However, static cold storage is still the preferred method for transplants from living donors because it is simple and effective at decreasing cellular aerobic metabolism. In this study, the authors aimed to analyze the short-term results of two static cold storage methods on graft function in kidney transplants from living donors.

**Methods:**

The authors prospectively assessed graft function in patients who underwent living-donor kidney transplantation between January 2020 and January 2023. Recipients were allocated to two preservation techniques by coin toss (ice bag vs. Raytech sponge). Given the marked allocation imbalance, all analyses were interpreted as exploratory and hypothesis-generating.

**Results:**

According to creatinine and eGFR measurements, postoperative day-7 and -30 creatinine levels in Group A were nominally lower (*p* < 0.05 and *p* < 0.05, respectively). There was no significant difference at postoperative days-60 and -180. The hospitalization time for Group A was shorter than for Group B (*p* < 0.05).

**Conclusions:**

The short-term biochemical outcomes of the ice bag and Raytech sponge techniques were broadly similar. Any early differences in creatinine/eGFR should be interpreted cautiously as exploratory findings that require confirmation in adequately powered, methodologically robust studies.

## Highlights

• This study compared two widely used static cold storage techniques in kidney transplantation.
• Both techniques yielded comparable short-term graft outcomes at six months.
• The ice bag technique showed a modest early advantage during the initial postoperative period.

## Introduction

Sustaining graft viability between extraction and implantation is crucial for optimal function. Currently, Static Cold Storage (S-CS) remains the most widely used preservation method. S-CS reduces Warm Ischemia (WI) time.^1^ WI, which is unavoidable during vascular anastomosis in kidney transplantation, may be linked to delayed graft function or nonfunction.^2^ Newer techniques like normothermic or hypothermic machine perfusion are succeeding in deceased donor transplantation. However, S-CS remains preferred in living donor transplantation due to its simplicity and ability to lower intracellular oxygen needs for aerobic metabolism.^3^ S-CS is also practical and cost-effective.^1^

The literature describes several strategies for Static Cold Storage (S-CS), including the Raytech sponge technique, the use of ice-soaked laparotomy pads, and the ice bag technique, which involves preserving the graft in an ice-filled plastic bag.^4^ These S-CS approaches have been assessed for outcomes including graft temperature, dysfunction, and Delayed Graft Function (D-GF) .^5^, ^6^, ^7^ Although several studies have examined the short-term results of these S-CS techniques, a lack of comparative data remains on early biochemical graft function, specifically in living-donor kidney transplantation.

This study prospectively compared the short-term outcomes of two S-CS techniques, Raytech sponge and ice bag, on graft function in living-donor kidney transplantation.

## Materials and methods

### Patient selection

This prospective comparative study with attempted random allocation was conducted at Istanbul Okan University Health Center, Transplantation Clinic, in Istanbul, Turkey. All participants underwent living-donor kidney transplants between January 2020 and January 2023. Candidate recipients for kidney transplantation were selected according to criteria described in the previous studies.^8^, ^9^, ^10^ All surgical procedures were performed by the same two transplant surgeons.

### Exclusion criteria

The study population was limited to living-donor kidney transplants, as prolonged cold ischemia time in deceased-donor transplantation may affect DGF and SGF rates.

To maintain surgical technique consistency, the authors excluded cases involving more than one artery or vein. Recipients who did not comply with follow-up protocol or were followed at other institutions were excluded.

### Surgical technique

The authors applied the classic extraperitoneal technique of the iliac fossa, as described in detail in recent papers.^11^^,^^12^ Following arterial and venous anastomosis and arterial perfusion of the graft, a double-J stented ureteroneocystostomy was completed using the Lich–Gregorin method.^13^

### Kidney preservation technique during vascular anastomosis

**Fig. 1:**
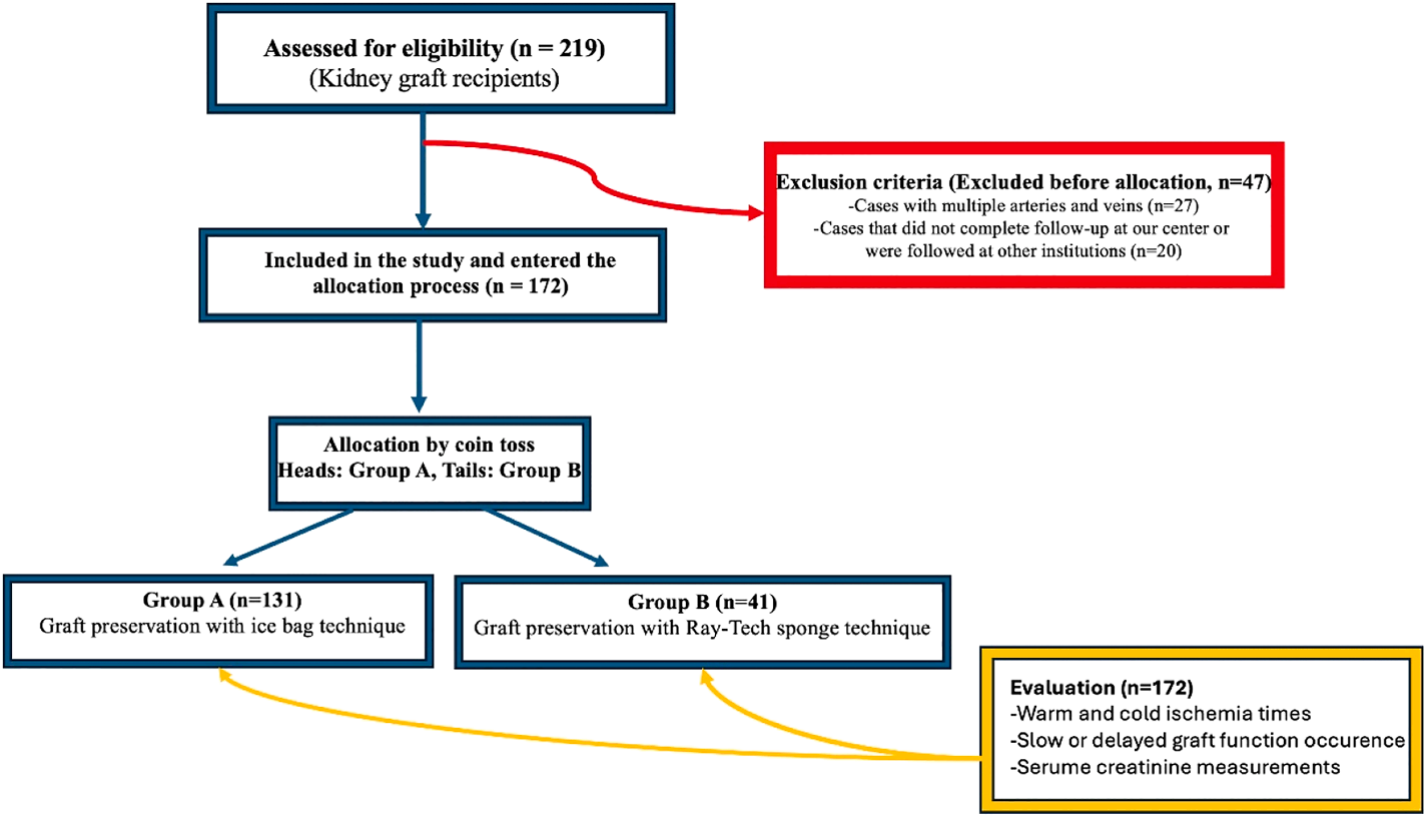
CONSORT flow diagram of the study.

The ice bag technique involves creating a small hole in the bottom-middle edge of the plastic bag, near the renal hilum, after back-table preparation. The graft is placed in the bag, and the vascular structures pass through that hole. The bag is refilled with Ringer's lactate slush to maintain cold ischemia. A Kocher clamp keeps fluid and ice inside. A Hartman Clamp marks the ureter side for orientation (Fig. 1).

### Raytech sponge technique

**Fig. 2:**
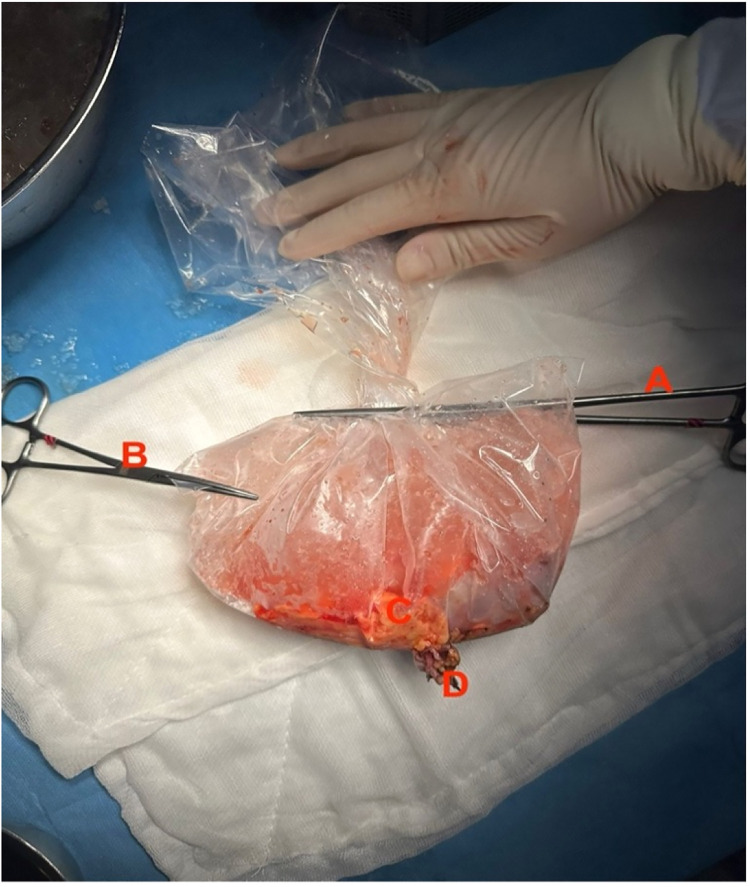
Ice-bag technique. (A) A Kocher clamp was used to keep the fluid and ice inside the plastic bag and to grip the graft, (B) Hartman-Mosquito Clamp was used to mark the ureter side to keep orientation, (C) Renal hilum, (D) Graft renal artery and vein.

**Fig. 3:**
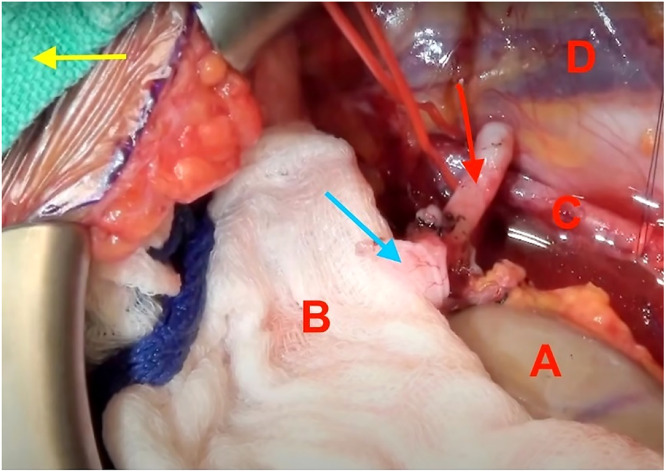
Raytech Sponge technique. Yellow arrow: Indicates cephalic side, Red arrow: Renal artery of the graft, Blue arrow: Renal vein of the graft. (A) Kidney, (B) Ice-soaked Raytech sponge, (C) External iliac artery, (D) Peritoneum.

Following the back-table preparation, a Raytech sponge is soaked in ice-cold lactated Ringer solution and covered around the graft. Orientation of the graft was maintained by direct vision of the ureter (Fig. 2, Fig. 3).

### Study groups and allocation procedure

Recipients were intended to be allocated to one of the two preservation groups by a coin-toss procedure after anesthesia induction. The allocation was performed by the circulating nurse independently of the surgical team. However, the final distribution was markedly imbalanced (Group A = 131; Group B = 41), which raises uncertainty as to whether the allocation procedure was implemented exactly as planned in every case. The exact mechanism underlying this discrepancy could not be determined from the available study documentation. Therefore, although a coin toss was the intended allocation method for all patients, undocumented deviations from the planned allocation procedure cannot be excluded.

Therefore, the authors report all findings as exploratory and hypothesis-generating. Primary analyses followed a modified intention-to-treat approach. A CONSORT-style flow diagram summarizing patient eligibility and allocation is provided in Fig. 1.

The result was communicated to the attending surgeon. No other personnel were involved or interfered during the randomization process. (Heads: Group A, Tails: Group B). Group A: Kidney transplantation with the ice bag technique. Group B: Kidney transplantation with the Raytech sponge technique. The CONSORT flow diagram is shown in Fig. 1.

### Immunosuppressant therapy protocol

The immunosuppressant protocol used at the studied institution is outlined in Table 1.

**Table 1:** Immunosuppressant therapy protocol applied at the studied institution.

	Tacrolimus^b^	Methylprednisolone	MMF^c^	ATG^d^	Prednisone
Day 1	0.1 mg/kg				
Day 0^a^	0.1 mg/kg	1000 mg	1440 mg	2.5 mg/kg	
Day 1	0.1 mg/kg	500 mg	1440 mg	2.5 mg/kg	
Day 2	Daily blood level test^b^	250 mg	1440 mg	2.5 mg/kg	
Day 3	Daily blood level test^b^	125 mg	1440 mg	2.5 mg/kg	
Day 4	Daily blood level test^b^	80 mg	1440 mg	2.5 mg/kg	
Day 5	Daily blood level test^b^	40 mg	1440 mg	2.5 mg/kg	
Day 6	Daily blood level testb		1440 mg		20 mg

a The operation day.

b The tacrolimus dose is adjusted according to blood tacrolimus level (8‒10 ng/mL).

c Sodium mycophenolate (384.8 mg).

d Anti-human T-lymphocyte immunoglobuline.

### Compared variables

The authors assessed demographics, WI (Warm Ischemia) and CI (Cold Ischemia) times, and occurrence of S-GF (slow graft function) or D-GF (delayed graft function).^14^^,^^15^ Serum creatinine and estimated Glomerular Filtration Rate (eGFR) were measured on postoperative days-7, -30, -60, and -180 after transplantation.^9^^,^^16^ Regarding proteinuria, the authors analyzed the sixth-month data to eliminate confounding factors, such as native kidney proteinuria.

### eGFR calculation

eGFR was calculated using the Modification of Diet in Renal Disease (MDRD) formula, which incorporates serum creatinine, age, sex, and race parameters. This method is widely used in clinical transplantation studies to assess renal function and provides a standardized estimation of glomerular filtration rate across patient populations.^17^

### Statistical analysis

Baseline characteristics and outcomes were compared between groups using appropriate tests according to data type and distribution. Categorical variables are presented as counts (%) and were compared using the chi-square test or Fisher's exact test, as appropriate. Continuous variables are presented as mean ± SD or median (IQR) and were compared using the Student's *t*-test for approximately normally distributed data or the Mann-Whitney *U* test for non-normally distributed data. Analyses were conducted on a modified intention-to-treat basis.

For binary outcomes, the authors calculated absolute risk differences with 95% Confidence intervals. Serum creatinine was assessed at prespecified postoperative follow-up time points (days-7, -30, -60, and -180); postoperative day-1 values were considered perioperative and are reported descriptively. Between-group comparisons at each follow-up time point are reported as exploratory; nominal two-sided *p*-values are provided, and the risk of type I error due to multiple comparisons is acknowledged. Where applicable, multiplicity-adjusted *p*-values (Bonferroni) for the prespecified follow-up time points are provided in Supplementary Table 5. The *p*-values are presented for descriptive purposes with a nominal reference threshold of *p* < 0.05 and should not be interpreted as confirmatory evidence. All analyses were performed using Minitab (Pennsylvania, USA).

### Ethical approval

The institutional review board of Istanbul Okan University approved the research protocol (n° 201, Date: April 21, 2020). This trial was designed and conducted in accordance with the principles outlined in the Helsinki Declaration. All participants provided written informed consent prior to enrollment in the study. Additionally, this study is publicly available on ClinicalTrials.gov (NCT06611189).

### Data availability statement

The datasets generated and/or analyzed during the current study are not publicly available due to institutional and ethical restrictions regarding patient confidentiality, but are available from the corresponding author upon reasonable request and with appropriate institutional approvals.

### Power analysis

A priori power analysis was performed with G*Power (v3.1.9.2) to estimate the required sample size. The study power was set at 80% (type II error probability = 20%). Based on prior literature,^5^^,^^6^^,^^8^, ^9^, ^10^ the expected combined D-GF and S-GF rate was estimated to yield an effect size of approximately *d* = 0.5‒0.7. To reach a 20% type II error (80% power) at a 0.05 level, at least 37 recipients were needed in each group. However, observed event rates in the studied cohort were much lower than expected. This meant the study was underpowered to detect small differences between groups. Therefore, the power analysis is presented for transparency, not as a guarantee of adequate power.

## Results

A total of 221 kidney transplants were performed between January 2020 and January 2023 at the studied institution. During the primary radiological assessment, 27 cases were excluded due to multiple arteries and veins. In addition, 20 cases were excluded for not following their follow-up program or for being followed at another institution. Two cases were excluded due to graft loss in the first six months after transplantation. As a result, 172 cases were eligible for the study protocol. Table 2 presents the demographics and etiologies of chronic kidney disease in these cases. When comparing ischemia and total surgery times between groups, both CI and total surgery times were longer in Group B compared to Group A; however, this difference was not statistically significant (Table 3).

**Table 2:** Demographics and CKD etiologies.

	Group A (*n* = 131)	Group B (*n* = 41)	a*p*-value
Demographics			
Age (years), Mean ± SD (Min–Max)	41.3 ± 12.6 (12–69)	41.6 ± 12.7 (21–67)	0.89
Gender (Male/Female), *n*	67/53	22/15	0.85
Height (cm), Mean ± SD (Min–Max)	164.1 ± 6.9 (149–201)	159.3 ± 10.4 (141–187)	0.07
Weight (kg), Mean ± SD (Min–Max)	65.2 ± 7.8 (31–127)	71.2 ± 9.8 (43–101)	0.001
BMI (kg/m^2^), Mean ± SD (Min–Max)	24.3 ± 4.2 (18.2–41.7)	27.4 ± 3.7 (19.4–40.1)	<0.001
Family History of CDK, *n* (%)	41 (34.1)	8 (21.6)	0.16
Smoker, ex-smoker, *n* (%)	51/13 (42.5/10.8)	15/6 (40.5/16.2)	0.67
CKD ethiology			
Diabetes mellitus, *n* (%)	61 (46.5)	17 (41.4)	
Hypertension, *n* (%)	33 (25.2)	13 (31.7)	
Amyloidosis, *n* (%)	3 (2.3)	2 (4.8)	
Goodpasture syndrome, *n* (%)	3 (2.3)	2 (4.8)	
Nephrolithiasis, *n* (%)	8 (6.1)	2 (4.8)	
Focal segmental glomerulosclerosis, *n* (%)	3 (2.3)	0 (0)	
Systemic lupus erythematosus, *n* (%)	3 (2.3)	1 (2.4)	
Polycystic kidney disease, *n* (%)	6 (4.5)	0 (0)	
Unknown etiology, *n* (%)	11 (8.3)	4 (9.7)	

CDK, Chronic Kidney Disease.

Etiology categories were compared descriptively because of small cell counts.

a Mann-Whitney *U* test, * *p* < 0.05; ** *p* < 0.01.

**Table 3:** Total surgery and ischemia times.

	Group A (*n* = 131)	Group B (*n* = 41)	a*p*-value
Warm ischemia time (minutes), Mean ± SD (Min ± Max)	1.9 ± 0.5 (1.5‒3)	2.1 ± 0.3 (1.5‒3)	0.49
Cold ischemia time (minutes), Mean ± SD (Min ± Max)	57.2 ± 10.1 (37‒74)	60.7 ± 24.6 (40‒71)	0.56
Surgery time (minutes), Mean ± SD (Min ± Max)	67.4 ± 13.8 (49–88)	71 ± 14.9 (52–91)	0.32

a Mann-Whitney *U* test, *p < 0.05; **p < 0.01.

**Fig. 4:**
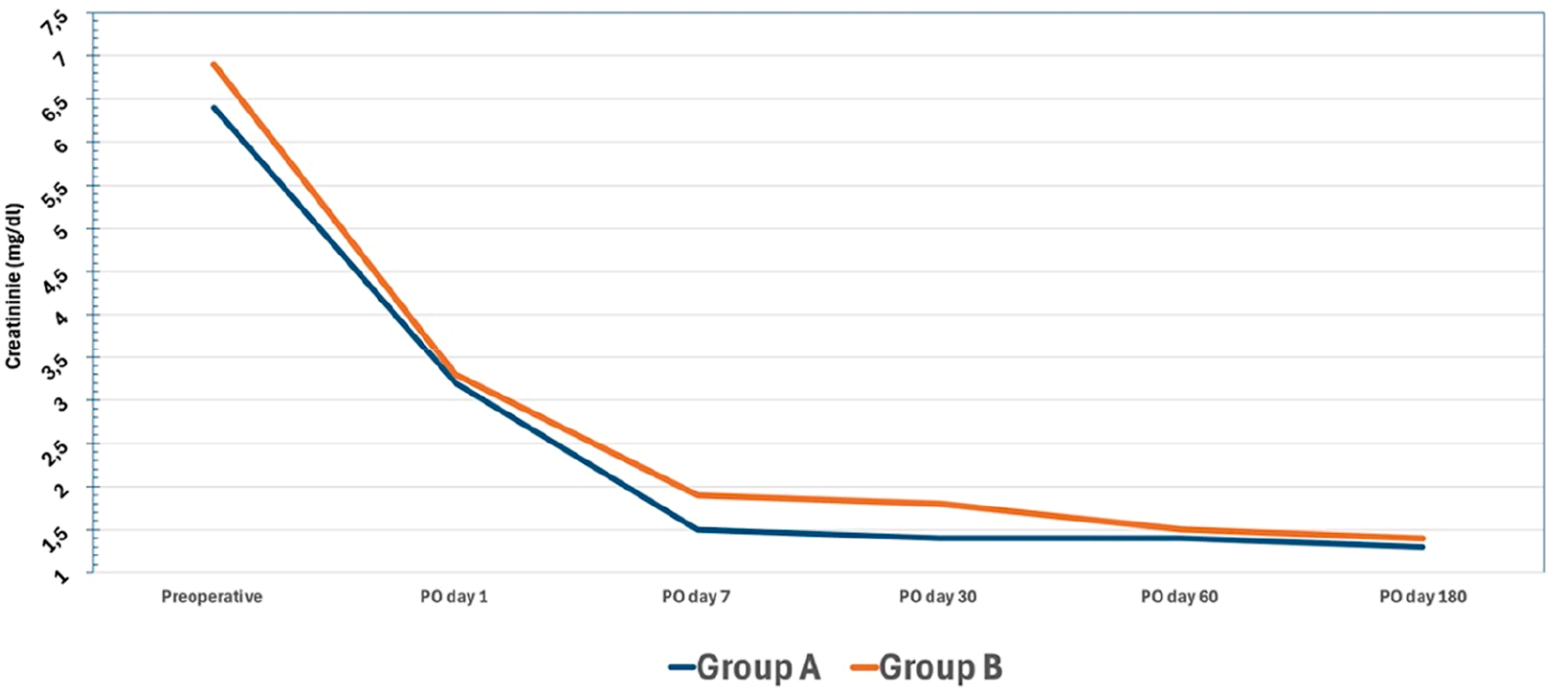
Creatinine decreases in Groups A and B.

Postoperative days-7 and -30 creatinine levels were nominally lower in Group A compared to Group B (*p* = 0.03 for both comparisons) (Table 4). Although these differences reached nominal significance, they should be interpreted cautiously as part of an exploratory, prespecified longitudinal analysis. There was no statistically significant difference between the groups at postoperative days-60 and -180 (Fig. 4).

**Table 4:** Creatinine, eGFR measurements, Delayed and Slow graft function, and hospitalization time.

	Group A (*n* = 131)	Group B (*n* = 41)	a*p*-value^b^
Preoperative creatinine (mg/dL), Mean ± SD (Min‒Max)	6.4 ± 3.2 (1.7–12.1)	6.9 ± 3.1 (1.6–11.7)	0.26
Postoperative day-1 creatinine (mg/dL), Mean ± SD (Min‒Max)	3.2 ± 1.8 (0.8–9.8)	3.3 ± 2.1 (1.1–7.8)	0.36
Postoperative day-7 creatinine (mg/dL), Mean ± SD (Min‒Max)	1.5 ± 1.0 (1.2–4.0)	1.9 ± 0.5 (1.4–3.9)	*0.03*
Postoperative day-30 creatinine (mg/dL), Mean ± SD (Min‒Max)	1.4 ± 0.7 (0.7–4.1)	1.8 ± 0.4 (0.9–4.1)	*0.03*
Postoperative day-60 creatinine (mg/dL), Mean ± SD (Min‒Max)	1.4 ± 0.6 (1.1–3.9)	1.5 ± 0.3 (1.0–4.9)	0.44
Postoperative day-180 creatinine (mg/dL), Mean ± SD (Min‒Max)	1.3 ± 0.6 (0.9–4.4)	1.4 ± 0.4 (0.8–3.7)	0.46
Preoperative eGFR (mL/min/1.73m^2^), Mean ± SD (Min‒Max)	5.7 ± 1.8 (3–10)	7.8 ± 1.5 (6–10)	0.21
Postoperative day-1 eGFR (mL/min/1.73m^2^), Mean ± SD (Min‒Max)	36.7 ± 11.8 (27–51)	33.8 ± 10.5 (21–53)	0.34
Postoperative day-7 eGFR (mL/min/1.73m^2^), Mean ± SD (Min‒Max)	60.8 ± 9.7 (40–63)	59.3 ± 10.6 (33–66)	*0.04*
Postoperative day-30 eGFR (mL/min/1.73m^2^), Mean ± SD (Min‒Max)	62.2 ± 7.7 (43–69)	56.7 ± 8.2 (39–68)	*0.03*
Postoperative day-60 eGFR (mL/min/1.73m^2^), Mean ± SD (Min‒Max)	62.7 ± 8.4 (42–68)	64.1 ± 7.6 (43–69)	0.38
Postoperative day-180 eGFR (mL/min/1.73m^2^), Mean ± SD (Min‒Max)	67.8 ± 6.7 (47–73)	68.3 ± 5.9 (41–76)	0.47
Delayed graft function, *n* (%)	3 (2.2)	1 (2.4)	0.96
Slow graft function, *n* (%)	6 (4.5)	3 (7.3)	0.49
Hospitalization time (days) Mean ± SD (Min‒Max)	7.49 ± 3.72 (6–21)	9.2 ± 4.9 (4–32)	*0.04*

All serum creatinine measurements were performed using the same Jaffe compensated method (Siemens Advia Chemistry System). The low SD values in Group B at postoperative days-60 and -180 should be interpreted with caution given the small sample size. The authors re-checked the underlying data and calculations and found no evidence of a data-entry or computation error; nonetheless, this may reflect limited variability within this subgroup.

a Continuous variables: Student’s *t*-test or Mann–Whitney *U* (depending on distribution), Categorical variables: Fisher’s exact test (or χ^2^ where appropriate).

b *p*-values are unadjusted for multiplicity and should be interpreted as nominal.

In the assessment of eGFR, Group A showed a modest trend toward higher eGFR values at days-7 and -30 (*p* = 0.04 and *p* = 0.05, respectively). No meaningful difference was observed between the groups at postoperative days-60 and -180 (Table 4).

Proteinuria at month six was analyzed to eliminate confounding factors, such as native kidney proteinuria. Mean proteinuria was 0.49 ± 0.25 g/24 h (range 0.11–0.47) in Group A and 0.52 ± 0.35 g/24 h in Group B, with no meaningful difference (*p* > 0.05).

Regarding early graft function, the rates of slow and delayed graft function were numerically higher in Group B, and the mean hospitalization time was shorter in Group A (Table 4 and 5).

**Table 5:** Bonferroni-adjusted *p*-values for creatinine and eGFR across postoperative time points (days-7, -30, -60, and -180).

Outcome	Day 7 *p*-value	Day 7 *p*-value adjusted	Day 30 *p*-value	Day 30 *p*-value adjusted	Day 60 *p*-value	Day 60 *p*-value adjusted	Day 180 *p*-value	Day 180 *p*-value adjusted
Creatinine	0.03	0.12	0.03	0.12	0.44	1.00	0.46	1.00
eGFR	0.04	0.16	0.03	0.12	0.38	1.00	0.47	1.00

Bonferroni adjustment was applied across the prespecified postoperative follow-up time points for creatinine and eGFR (days-7, -30, -60, and -180; m = 4). Earlier perioperative measurements were not part of this prespecified follow-up schedule.

## Discussion

The present study showed that creatinine levels decrease more rapidly in the early stages of kidney transplantation using the ice bag technique. However, two months after graft implantation, creatinine levels reached the same level in both methods.

Graft preservation and perfusion methods are among the greatest challenges in kidney transplantation. Acute interruption of blood flow causes a significant decrease in aerobic metabolism, leading to hypoxia and ischemic cell injury. However, rapid revascularization can provoke ischemia-reperfusion injury.^18^ In response, the inflammation cascade is triggered. This leads to pathological lesions like endothelial dysfunction, increased vascular permeability, apoptosis, and cell necrosis.^19^ Clinically, these manifest as S-GF or D-GF, and infrequently as non-functional grafts.^20^ In the present study, delayed and slow graft function rates are higher in the Raytech sponge group; however, the difference is not statistically significant. Potential explanations for this numerical (non-significant) trend include residual confounding or chance; given the lack of statistically significant differences in ischemia times, the authors avoid causal attribution. These findings are consistent with previous studies.^4^^,^^15^^,^^21^

Over the past two decades, technological advances have driven major developments in organ preservation. Normothermic and hypothermic machine perfusion techniques have improved graft function and survival, especially in deceased donor transplants.^22^ However, these techniques are not routinely used in living donor transplants due to technical difficulties, minimal cold ischemia time, and lack of cost-effectiveness. The Raytech sponge and ice bag techniques are simple, cost-effective alternatives.^23^ Thus, these two static cold storage techniques offer practical advantages over more complex systems in living donor transplantation.

Ortiz et al. assessed the efficiency of the ice bag technique and found the D-GF rate to be 20% in their cohort.^2^ A 2019 Cochrane database analysis found that delayed graft function was 13.6% in grafts preserved with S-CS.^7^ Both studies were conducted using grafts from deceased donors. In contrast, the present study utilized grafts from living donors, making it a unique approach. In the present cohort, the D-GF rate in both groups was lower than that reported in the literature. Consistent with other reports,^24^^,^^25^ the authors attribute this difference to the surgical team's experience and the short hot-cold ischemia times resulting from that experience.

Dergham et al. described a new graft cooling device in an experimental study using a pig model, published in 2024. They called ice bag techniques cumbersome and clumsy, claiming these methods prevent adaptation during surgery.^26^ In their study, only graft external and core temperatures were measured, and the new device was stated to be superior. However, graft functions were not evaluated, raising doubts about its clinical use. This study stands out for its focus on short-term renal function, utilizing biochemical markers. Nonetheless, further prospective randomized studies are warranted, especially those correlating graft core temperature with biochemical, histomorphological, and radiological findings.

Numerous studies have demonstrated that proteinuria is a predictor of long-term graft survival after transplantation.^27^ The present study shows there is no difference in proteinuria at the sixth month after graft implantation in either static cold storage technique. These results suggest that proteinuria at six months is similar between techniques; however, longer follow-up is needed to assess long-term graft survival. However, larger and longer studies that include histomorphological evaluation are needed to confirm this finding.

The number of studies on graft preservation is rising.^1^, ^2^, ^3^, ^4^, ^5^, ^6^, ^7^ Recently, static (cold ischemia) and dynamic (normothermic and hypothermic machine perfusion) techniques have been compared. The benefits of dynamic preservation techniques for graft function and long-term outcomes are well documented.^7^ However, these approaches may be costly and technically demanding, particularly in living-donor transplantation. Therefore, Static Cold Storage (SCS) remains widely used in many centers because of its simplicity and cost-effectiveness.^28^^,^^29^ Ide et al. reported that a thermal barrier bag could help maintain lower renal temperatures during vascular anastomosis, aiming to limit warm ischemic exposure during implantation.^30^ In contrast, this study compared two practical SCS approaches used during implantation to maintain graft cooling. The authors observed a modest, exploratory early postoperative trend toward lower serum creatinine and higher eGFR in the ice bag group, while six-month renal function was comparable between techniques; these early differences should be interpreted cautiously given the allocation imbalance, limited statistical power, and multiple comparisons.

In the present study, serum creatinine appeared numerically lower in the ice bag group on postoperative days-7 and -30, and eGFR showed a similar numerical pattern at these time points. The authors acknowledge, however, that warm ischemia time did not differ significantly between groups (Table 3), and given the allocation imbalance, limited statistical power, and multiple comparisons, these observations should be interpreted with considerable caution and viewed as exploratory. Therefore, the authors do not wish to imply any causal relationship (including an effect mediated by warm ischemia), and these early trends should be confirmed in larger, adequately powered studies.

### Limitations of the study

This study has several limitations. First, it was conducted at a single center with a relatively small sample size, and thus, larger multicenter studies are required to validate the findings. Second, because all participants were of Caucasian ethnicity, the generalizability of the results to other kidney transplant populations may be limited. Third, the use of non-standard immunosuppressive treatment in a small number of patients represents another potential source of heterogeneity. A further important limitation relates to the allocation process. Although patients were intended to be assigned by a coin toss performed by the circulating nurse, the marked 3:1 imbalance between groups raises uncertainty regarding whether this procedure was strictly followed in every case. The available study documentation does not allow us to determine whether the observed imbalance reflected undocumented deviations from the intended allocation procedure in some cases. Therefore, strict adherence to the intended allocation method cannot be confirmed, selection bias cannot be excluded, and the internal validity of the comparisons between groups is limited. In addition, some baseline demographic characteristics, particularly height, weight, and BMI, differed between the groups, which may have introduced residual confounding. Although propensity score methods were initially planned, they were not performed due to the small sample size and marked imbalance, which limited their reliability. Excluding grafts with multiple arteries or veins also restricted generalizability, since these are common in clinical transplantation. However, this criterion ensured population homogeneity and avoided confounding surgical factors. Observed event rates were lower than expected, indicating that the study lacked sufficient power to detect small differences between groups. Thus, the authors present the power analysis for transparency. The low rates of delayed and slow graft function may be related to the use of living donors, short cold ischemia times, and minimal ischemia-reperfusion injury. Another limitation is that the authors only focused on graft function and did not include histomorphological analyses. Other factors, such as inotropic agent use, donor serum creatinine, hypovolemia history, and donor BMI, were not evaluated. Also, early postoperative proteinuria may have been affected by native kidney function, potentially leading to misleading results. Therefore, only the six-month proteinuria data were analyzed. Assessing the effects of both static techniques on iohexol or iothalamate clearance would have been scientifically valuable; however, these tests are not routine in the present center and are costly and logistically challenging. Finally, although a Bonferroni adjustment was applied to repeated creatinine and eGFR comparisons, other analyses were not formally adjusted for multiplicity; therefore, these results should be considered exploratory.

## Conclusion

Living-donor kidney transplantation is associated with excellent outcomes and short cold ischemia times, highlighting the interest in simple, safe, and cost-efficient preservation approaches. In this prospective comparative cohort with attempted random allocation, the two static cold storage techniques were associated with similar six-month graft outcomes. Although the ice bag technique showed numerically more favorable early postoperative biochemical values, these differences are exploratory and hypothesis-generating only and should not be interpreted as confirmatory evidence of benefit or superiority.

Interpretation of these findings is substantially limited by the marked allocation imbalance and low event rates, and the very low incidence of delayed or slow graft function reduced the authors’ ability to detect small between-group differences. In addition, the external validity of the study is limited, as the cohort was predominantly Caucasian and grafts with multiple vessels were excluded. Therefore, these results should be viewed cautiously and primarily as preliminary observations that require confirmation.

Overall, the present data do not support definitive clinical conclusions regarding the superiority of either method. Larger, multicenter studies with rigorous allocation procedures, appropriate control for multiplicity, broader patient and graft inclusion, and longer follow-up are needed before any generalizable clinical recommendations can be made.

## Authors’ contributions

Murat Ferhat Ferhatoğlu: Conceptualization; methodology; investigation; data curation; formal analysis; writing-original draft. Taner Kivilcim: Investigation; data curation; writing-review & editing. Sibel Y. Ferhatoglu: Supervision-revised draft; writing-revised draft. Alp Gurkan: Conceptualization; supervision; project administration. All authors read and approved the final version of the manuscript.

## Conflicts of interest

The authors declare no conflicts of interest.

## Appendix Supplementary materials

Image, application 1

Image, video 2 dummy alt text
