# Supplementary material for: An exploratory comparison of the short-term effects of two different static cold storage techniques of the graft in kidney transplantation from living donors
Source: Clinics (Sao Paulo). 2026 Jul 28;81:101061. doi: 10.1016/j.clinsp.2026.101061 (PMC13446367; doi:10.1016/j.clinsp.2026.101061)
Supplement: Supplementary file 1 [file mmc1.docx]

**CLINICS-D-25-01351_Supplementary Material**

**Video 1** Demonstration of graft placement in the ice-bag technique.
